# Effect of Nutritional Flushing Using Long-Term Energy and Protein Supplementation on Growth Performance and Reproductive Parameters of Doyogena Ewes in Ethiopia

**DOI:** 10.3390/vetsci10060368

**Published:** 2023-05-23

**Authors:** Asfaw Tesfaye, Bimrew Asmare, Tesfaye Abiso, Jane Wamatu

**Affiliations:** 1 Department of Animal Sciences, Bahir Dar University, Bahir Dar P.O. Box 5501, Ethiopia; 2 Southern Agricultural Research Institute, Areka Agricultural Research Center, Addis Ababa P.O. Box 5689, Ethiopia; 3 International Center for Agricultural Research in the Dry Areas (ICARDA), Addis Ababa P.O. Box 5689, Ethiopia

**Keywords:** Doyogena ewes, flushing, body condition, reproductive performances

## Abstract

**Simple Summary:**

Nutritional flushing has the potential to increase reproduction in small ruminants. Utilization of locally available feed resources is imperative if smallholder farmers are to adopt improved nutritional strategies for flushing sustainably. The current experiment investigated the effect of four isonitrogenous (14.7% crude protein) rations formulated to supply low (Lo-ME) or high (Hi-ME) metabolizable energy based on enset leaf (*Ensete ventricosum*) and commercial concentrate and the effects on local Doyogena ewes in Ethiopia. The current finding indicated the supplementation of both enset leaf and concentrate enhanced the performance of local ewes.

**Abstract:**

The objective of the study was to establish the effect of appropriate supplementation days (days -21 to +7) using four isonitrogenous (14.7% CP) diets balanced to provide low (Lo-ME) or high (Hi-ME) metabolizable energy on the body condition score (BCS), body weight (BW) change, and reproductive performances of sheep. Thirty-five Doyogena ewes (27.71 ± 2.87 kg, 2–5 years of age, BCS of 2.0–2.5) grazing on natural pasture were randomly assigned to supplementary treatments consisting of combinations of enset leaf (EL) and commercial concentrate (CC): T0 (control), T1 (250 g EL + 500 g CC: Lo-ME), T2 (400 g EL + 500 g CC; Hi-ME), T3 (500 g EL + 400 g CC; Hi-ME), and T4 (500 g EL + 250 g CC; Lo-ME). The estrous cycle was synchronized with one intramuscular injection, 5 mg PGF2α, prior to artificial insemination. The dry matter (DM) from the pasture provided 1.10–1.46 kg/day, which corresponds to the DM requirements of the ewes until late gestation. However, the pasture provided a protein content of 9.52%, which was insufficient for breeding, mid-gestation, and gestation, requiring minimums of 16.1%, 13.1%, and 14.8%, respectively. The pasture could only provide enough energy for breeding ewes with a BW of up to 30 kg. The energy provided by pasture was insufficient for ewes weighing > 30 kg at mid-gestation and gestation, providing 6.9–9.2 MJ/day, below the requirement of 11.92–16.32 MJ/day required for mid-gestation and gestation. The energy was not sufficient for large ewes weighing > 40 kg. Supplementary diets T1–T4 provided DM in the range of 1.7–2.29 kg/day. This was sufficient for AI, mid-gestation, and gestation phases. Dietary supplements increased (*p* < 0.01) BW during breeding and mid-gestation. During lambing, T2 and T3 increased BW (*p* < 0.05) compared to T4 and T1. T4 had a similar effect (*p* > 0.05) on BW during lambing. T1, T2, and T3 significantly increased BCS (*p* < 0.05). T2 and T3 increased (*p* < 0.05) BCS at mid-gestation, but only T2 significantly increased BCD (*p* < 0.05) during lambing. All dietary supplements resulted in a shorter (*p* < 0.05) time to the resumption of estrous and the length of estrous (*p* < 0.05). T1, T2, and T3 resulted in a stronger estrous response (*p* < 0.05). Dietary supplements enhanced (*p* < 0.05) the conception rate and fecundity rate. The conception rate was highest in T2 and T3 at 85.7% and 83.3%, respectively. T2 had the highest fecundity rate at 151.7% (*p* < 0.05). Dietary supplementation increased the rate of lambing (LR), litter size (LS), and weight of lambs at birth (LBW). The LR for treatments T2, T3, and T4 was 100% versus 66.7% in the control. T1 and T2 significantly increased (*p* < 0.05) LS, but T4 had a similar LS to the control. Dietary supplements T1, T3, and T4 tended to increase (*p* < 0.05) LBW, but T2 increased LBW significantly (*p* < 0.05). Supplementation (T2, T3) with 400 g enset + 500 g CC and 500 g enset + 400 g CC are promising feed supplements to increase the reproductive capacities of Doyogena ewes in Ethiopia. Energy is as important to ewe flushing as protein.

## 1. Introduction

Ethiopia is characterized by crop–livestock agriculture. Sheep are important for the national and household economy. The country has 39.89 million sheep; of this, approximately 70.05% are indigenous breeds [1]. Sheep are an important income source, a source of food, and serve as a raw materials source for industry. Despite the large population of sheep, their performance per individual animal and population is hampered by inadequate and poor-quality feed, disease and parasites, underdeveloped infrastructure, and the lack of organized breeding programs and policies. Improved reproductive performance of sheep is crucial to realize the potential of the sector in the country. It has been reported that the reproductive parameters of small ruminants, including sheep, are positively related to feeding management, particularly energy balance [2]. Increased energy intake during the early breeding season is essential to improve reproductive performance. One of the methods of improving the reproduction performance of sheep is flushing. Nutritional flushing increases the number of blood metabolites, such as glucose, which may play a vital role in folliculogenesis and ovulation processes [3,4]. Depending on the animal’s energy level, neuroendocrine cells are produced to control the secretion of gonadotropin-releasing hormone release [5]. This consequently affects follicular development and ovulation.

Additionally, improving the quality of feeds and feeding practices of livestock is an essential strategy to improve ovulation rate and reproductive efficiency in animals. In sheep, nutritional status, adequate energy balance, and protein absorption are key factors that regulate ovulation rate and overall reproductive performance. The interface between reproduction and nutrition in sheep has been reported by ref. [6]. Previous studies of similar types of animals indicated flushing enhances ovulation and uterine implantation of the fetus [7]. Livestock flushing around the breeding season has attracted attention from sheep farmers to boost ovulation, conception rates, and the total number of lambs born. The Doyogena breed is regarded highly in Ethiopia for its high growth performance and reproductive capacity. However, flushing to increase reproductive performance in Doyogena ewes is yet to be studied.

Increasing energy and protein availability improves ewes’ reproductive efficiency [8]. Ewes should be flushed 2–3 weeks before and during breeding. Farmers rely on commercial concentrate to supplement livestock productivity. However, the high cost of concentrates, limited access, and availability limit it as a viable option. To promote the sustainable uptake of improved flushing strategies, it is imperative to develop feeding strategies based on locally available feed resources that are easily accessible to farmers. Doyogena ewes are predominantly found in the south and southern regions of Ethiopia. In these regions, enset (*Ensete ventricosum)* is a dominant crop, especially in the altitude range between 1400 and 3000 m above sea level. It also grows wild in parts of central, eastern, and southern Africa. Enset is a drought-tolerant herbaceous banana-like plant, also known as a false banana.

In the study area, livestock producers feed their animals with leaves from the enset plant alone or as a supplement to crop residue feeds during prolonged dry spells. Due to diminishing pasture land, the usage of enset leaves and other types of forages has increased over time. The high crude protein (CP) concentration in the lamina part of enset leaves (209 g/kg DM [9] makes it a valuable fodder for livestock. A mixture of enset leaf lamina and leaf midrib with an average CP content of 130 g/kg DM [10], enset leaf can be considered a useful protein fodder for livestock. The optimal dietary supplementation of energy and protein beyond main farmers‘ practices that could enhance the reproduction parameters of Doyogena ewes has not been elucidated. Energy and protein amounts required to enhance the reproductive parameters of Doyogena ewes are not yet determined in the study area. To the authors’ knowledge, there is no information on the feed value of enset leaf supplementation to improve reproductive performance in ewes. This study aimed to determine the effect of long-term nutritional supplementation with four isonitrogenous (14.7% CP) diets formulated to provide low (Lo-ME) or high (Hi-ME) energy based on the effects of enset leaf (*Ensete ventricosum*) and commercial concentrate on the body condition score (BCS), change in body weight (BW), and reproduction performances of Doyogena ewes.

## 2. Materials and Methods

### 2.1. Study Area

**Figure 1:**
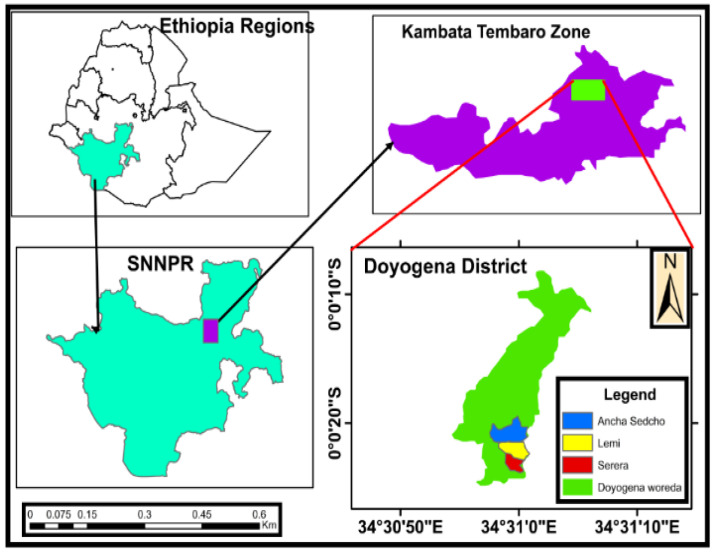
Map showing Doyogena District.

Doyogena District, located in the Southern Nations, Nationalities, Peoples Regional State (SNNPRS), Ethiopia, was the study site (Figure 1). It lies 258 km southwest of Addis Ababa, 1900–2748 m above sea level (m.a.s.l), with a latitude of 7°18′25″ N–7°21′49″ N and longitude of 37°45′33″ E–37°48′51″ E. The district covers an area of 17,263 hectares. The topography is moderately sloping (hilly to undulating to plains), partly plateau, and has a rugged terrain slope, with flat to gentle sloping plains. There are two rainy seasons, from June to September and from February to April. Temperatures range between 10 °C and 16 °C, and the mean annual rainfall is 1400 mm. Natural pastures consist of varying proportions of desho grass (*Pennisetum pedicellatum*) and elephant grass (*Pennisetum purpureum*). Doyogena has a mixed crop–livestock farming system [11]. The dominant crops in the highlands are enset, barley, cabbage, faba beans, potatoes, field pea, and wheat. At lower altitudes, farmers grow maize and sugar cane. 

### 2.2. Farmer Selection

The experiment was conducted under farmers’ conditions on-farm. Three peasant associations (PAs), namely Lemo, Serera, and Anicha Sedicho, were chosen based on the existence of adequate sheep numbers in the PA, the PA’s participation in the community-based breeding program (CBBP), a program initiated by the International Center for Agricultural Research in Dry Areas (ICARDA), farmer interest, farmer willingness to participate, and farmer ownership of more than six sheep. A total of 12 willing farmers from six distinct villages within the PAs were selected. Subsequently, six non-pregnant ewes were identified from each farmer’s flock. From these ewes, 35 Doyogena ewes were selected for the experiment based on the similarity of age, BW, and BCS. 

### 2.3. Experimental Animals and Their Management

The initial age of the ewes was between 2 and 5 years old, weight between 24 and 36 kg, and body BCS between 2 and 3. The ewes used were not pregnant, and they were dry animals. They had lambed during the lambing season preceding the study. All ewes were housed individually and kept visually and physically isolated from breeding rams. Ewes were ear-tagged and drenched against internal and external parasites using antihelminth albendazole (7.5 mg/kg weight, taken orally). Ivermectin was used for prophylaxis and control of external parasitism (was administered by subcutaneous injection (0.2 mg/kg weight). The broad-spectrum antibiotic (Oxytetracycline long act) was injected as a prophylaxis against secondary bacterial infections. Ewes were vaccinated against ovine pasteurellosis, sheep pox, peste des petits ruminants, anthrax, foot-and-mouth disease, and enterotoxemia. Animal care, handling, and maintenance during the experiment complied with Bahir Dar University Animal Welfare Regulations.

### 2.4. Experimental Feed Preparation and Management

Ewes grazed freely on natural pasture for at least 6 h/day and were supplemented with chopped enset leaf (EL) and commercial concentrate (CC) offered in equal amounts daily morning (8 a.m.) and evening (5 p.m.). Flushing started simultaneously in all groups with a 15-day adaptation period. Enset leaf and CC were provided in separate feeding troughs. Ewes had access to clean water and commercial salt licks *ad libitum*. Enset leaf (leaf lamina and leaf midrib) was harvested daily from mature enset plants of unselected varieties from local farmers. It was chopped with a knife (to a size of 1–10 cm) and prepared every late afternoon (after 5 p.m.) for feeding the next day. It was provided fresh daily. The CC mixture, with a labeled composition of 35% wheat bran, 30% noug seed cake, 35% coarsely ground maize grain, and 1% salt, was purchased from the John Farm animal feed processing factory in Hosanna Town and stored dry in a cool storage room pending incorporation into the experimental ration. The nutrient composition of the supplementary feed is shown in Table 1. 

### 2.5. Experimental Steup and Treatment Diets

The experimental design used was a seven-replicate randomized complete block design (RCBD). The ewes were divided into seven groups on the basis of their initial BW and randomly allocated to one of the five treatments consisting of EL and CC in various proportions. The treatments and their nutritional content at ewe bodyweights of approximately 30 kg (initial and during insemination) and BW of approximately 40 kg (mid-gestation and lambing) are shown in Table 2. Supplementation was provided 21 days before AI, and supplementation was continued for one week (7 days) after AI. At the end of the breeding season, ewes were kept on the same feeding plan during the gestation period.

### 2.6. Estrous Synchronization

To synchronize the onset of estrous, all ewes each received one intramuscular injection of 5 mg prostaglandin hormone (PGF2α analogue dinoprost (1 mL Enzaprost^®^; CEVA laboratories, Libourne, France) on Day 21 of nutritional flushing according to the protocol of ref. [12], modified for the Areka Agricultural Research Institute [13]. Thereafter, for two days (48 h), the ewes were observed three times a day for one hour by trained observers from 9:00 a.m. to 10:00 a.m., in the afternoon from 2:00 p.m. to 3:00 p.m., and for behavioral manifestations of estrous in the evenings from 6:00 p.m. to 7:00 p.m. After hormone injection and prior to artificial insemination (AI), aproned Doyogena-proven rams (1 ram per 3 ewes) were used as part of the behavioral stimulus of the teaser ram effect to synchronize estrous in the ewes [8]. Ewes were in estrous when they “stood to be mounted” by teaser rams. Other indications included active tail movement, an inflamed and red vulva, discharge of transparent mucus, restlessness, frequent bleating, and frequent urination. To inseminate, fresh semen was obtained using an artificial vagina (AV) and promptly examined for characteristics such as volume, appearance (including color and contamination), concentration, and motility. After screening for normality, the ejaculates were examined for sperm concentration, morphology, and sperm motility. Ejaculates that qualified were diluted to a final concentration of 400 × 10^6^ sperm in each straw (straw volume 0.25 mL) using a commercial sheep semen extender (Ovixcell; IMV^®^, France) kept at 35–37 °C. Ewes were inseminated with fresh extender semen using a vaginal speculum fitted with a white LED light and QuicklockR guns covered with a minitub sheath. The mean time elapsed between semen gathering and insemination was no higher than 10–12 min. Each ewe was artificially inseminated once. It was assumed that ewes were pregnant when lambing took place 150 ± 5 days after the day of insemination. The procedure for insemination was conducted according to ref. [13].

### 2.7. Data Collection

The study was undertaken for 185 days, including a gestation period of 150 days. Body weight, BCS, and reproductive parameters were recorded during the experiment.

#### 2.7.1. Body Weight Measurements

Body weight was measured at the initial stage of the study and once weekly until ewes were lambed. Measurements were taken at 8 a.m. after overnight fasting and before morning feeding and watering. A portable spring-dial hoist scale (Camry, NTB, Camry company, Zhongshan, China), with a capacity of 100 kg and an accuracy of 0.05 kg, was used. The scale was calibrated using standard weights. Ten sheep were weighed in three replicates to ascertain the reliability of the BW measurements. Weights of ewes were also recorded prior to and after parturition to determine loss of weight from lambing.

#### 2.7.2. Body Condition Score

The BCS of ewes was assessed on a scale of 1 to 5 through the examination of the muscling and fat deposition surrounding and over the vertebrae in the loin by palpation, as described by ref. [14]. This was recorded at the beginning of the trial, during AI, mid-gestation, and during lambing. 

#### 2.7.3. Reproductive Traits 

The present study evaluated the following data on estrous and reproductive performance:

▪ Estrous response (%) was determined by the number of ewes displaying standing estrous in each treatment group in relation to the total number of ewes.
▪ The onset of estrous was defined as the midpoint between the last rejection and first acceptance by a mounting male, while the end of estrous was determined as halfway between the last acceptance and first rejection [15].
▪ The duration of estrous was measured in hours, from the first to the last occurrence of standing estrous.
▪ Conception rate (%) was calculated as the number of pregnant ewes divided by the number of ewes that exhibited estrous and were inseminated, multiplied by 100.
▪ The lambing rate (%) was calculated as the number of ewes that gave birth divided by the number of pregnant ewes multiplied by 100.
▪ The fecundity rate was calculated as the number of lambs born divided by the number of ewes inseminated, multiplied by 100.
▪ The gestation period was defined as the duration between conception and parturition.
▪ Litter size at birth was calculated as the number of lambs born divided by the number of ewes that gave birth.
▪ The abortion rate was calculated as the number of ewes that were aborted divided by the number of ewes that were inseminated, multiplied by 100.

Birth weight was recorded shortly after birth within 24 h.

### 2.8. Chemical Analysis of Feeds

Sub-samples of 500 g representative EL and CC samples were collected twice weekly and pooled over a month and a half. For each collection, sub-samples of EL were stored in a freezer until they were processed for analysis. CC samples were stored in a cool, dry place until analyzed. A total of 5–75 snip samples, representative of herbage eaten by sheep, were collected randomly by hand at weekly intervals from each participating farmer’s homestead [16]. A subsample was sorted to remove dead and senescent material and broadleaf weeds and then dried to constant weight in an oven at 60 °C. Samples were placed in paper bags pending analysis. After oven drying at 100 °C for 24 h, all samples (EL, NP, CC, and treatment mixtures) were ground to pass through a 1 mm sieve mesh and analyzed using the standard wet chemistry method. Dry matter was determined by oven drying at 105 °C overnight (Method 934.01). Ash was determined by burning overnight in a muffle furnace at 500 °C (Method 942.05). Nitrogen content was determined by the Kjeldahl method using Kjeldahl (protein/nitrogen) Model 1026 (Foss Technology Corp.) (Method 954.01). A conversion factor of 6.25 was used to convert nitrogen to crude protein. Neutral detergent fiber, acid detergent fiber (ADF), and lignin were determined as described by ref. [17]. The neutral detergent fiber was expressed as exclusive of residual ash. The acid detergent fiber was expressed without residual ash. Lignin was determined by the solubilization of cellulose with sulphuric acid. In vitro, organic matter digestibility was measured in rumen microbial inoculum using an in vitro gas production technique. The buffer solution was prepared based on the procedures explained by ref. [18]. Rumen fluid was collected prior to morning feeding using a vacuum pump from three ruminally cannulated cows fed a total mixed ration of grass hay (790 g/kg), wheat bran (203 g/kg), salt (3.2 g/kg), and a mineral and vitamin mixture (4.6 g/kg) on a DM basis. The use of cows was assessed and approved by the Environmental and Occupational Health and Safety Unit of ILRI. The rumen fluid from the cows was composited (1:1, *v*/*v*), filtered through four layers of cheesecloth, and added to the buffer solution (1:2, *v*/*v*), which was maintained in a 39 °C water bath with continuous CO_2_ flushing. The buffered rumen fluid (30 mL) was pipetted into 100 mL syringes containing a 0.2 g sample and immediately placed in a water bath at 39 °C. Gas production was recorded after 24 h of incubation and used to calculate IVOMD according to ref. [19] using suitable equations for legume hays as follows:

IVOMD (g/kg) = 14.88 + 0.889GP + 0.45CP + 0.0651XA,

where GP: 24 h net gas production (mL/200 mg), CP: Crude protein (g/kg DM), and XA: Ash content (g/kg DM).

Metabolizable energy (ME) was estimated from digestible energy (DE) and IVOMD using regression and summation equations developed by ref. [20]: First, DE (Digestible energy) was obtained using the formula: 

DE = (0.01 × (OM/100) × (IVOMD + 12.9) × 4.4) − 0.3, then metabolizable energy could be calculated as follows: ME (Mcal/kg) = 0.82 × DE was calculated and converted to Kilogram ME (MJ/kg) = 4.184 × ME (Mcal/kg).

### 2.9. Data Analysis

The data collected in this experiment included BW and BCS at the onset of estrous, duration of estrous, LBW, LS, and pregnancy period, which were analyzed based on the GLM procedure of SAS version 9.2 in a completely randomized design. The Duncan multiple range test was utilized to differentiate treatment means. Chi-square categorical analysis was conducted for estrous response, conception, lambing, and fecundity rates. Within treatment groups, means were compared using general linear model (GLM) procedures. A *p*-value of <0.05 indicated a significant difference in mean differences, while 0.05 < *p* < 0.10 indicated a statistical tendency. The following model was used for data analysis: Yij = μ + Ti + Bj + eij. Where: 

Yij = dependent variables (onset of estrous, conception rate, lambing rate, litter size);

μ = the overall mean;

Ti = treatment (nutritional feeding) effect;

Bj = block (BW and BCS effect);

eij = random error.

## 3. Results

### 3.1. Effects of Nutritional Flushing on Body Weight of Doyogena Ewes 

Body weights recorded at the beginning of flushing (27.14–28.14 kg), at mating (29.14–31.86 kg), mid-gestation (34.93–37.64 kg), and lambing (31.14–32.79 kg) are shown in Table 3. Supplementary diets T1–T4 provided DM between 1.7–2.29 kg/day. This was sufficient for breeding, mid-gestation, and gestation phases, with a DM requirement of 1.18–1.48 kg/day. The findings elucidate that there was a similarity (*p* > 0.05) in the average BW of the ewes at the onset of the study. This could be due to the fact that there was no random error in the allocation of experimental animals to the treatment groups. Provision of all supplemental diets increased (*p* < 0.01) BW at breeding and mid-gestation. At lambing, T2 and T3 increased BW (*p* < 0.05) compared to T4 and T1. T4 had no effect (*p* > 0.05) on BW at lambing. The effects of T2 and T3 were similar at breeding, mid-gestation, and lambing.

### 3.2. Effects of Nutritional Flushing on Body Condition Score 

The BCS of the ewes during breeding, mid-gestation, and lambing is shown in Table 4. The mean BCS of the ewes at the beginning of the allocation of treatments (Day-21) was similar among treatment groups (*p* < 0.05). The BCS during breeding (BCS-B) was significantly higher (*p* < 0.05) for T1 (2.9), T2 (3.07), and T3 (3.00) compared to T0 (2.57). There was a gradual increase in BCS from breeding to lambing in all treatment groups. T2 consistently showed the best BCS from the start of the study to lambing.

### 3.3. Effects of Nutritional Flushing on the Reproductive Performance 

#### 3.3.1. Effect of Nutritional Flushing on Estrous

The number of ewes showing standing estrous, relative to the total number of ewes in each treatment group, expressed as a percentage (estrous response, %), time to onset of estrous, and duration of estrous, is shown in Table 5. Dietary supplements resulted in a shorter (*p* < 0.001) time to onset of estrous and a longer duration of estrous (*p* < 0.001). T1, T2, and T3 resulted in a greater estrous response (*p* < 0.05). T2 showed the earliest estrous and had the longest estrous duration.

#### 3.3.2. Effect of Nutritional Flushing on Conception, Gestation, Abortion, and Fecundity 

The rates of conception, gestation, fecundity, and abortion are shown in Table 6. Dietary supplements increased (*p* < 0.001) conception rates and fecundity rates. The conception rate was highest in T2, T3, and T4 at 100%. Fecundity ranked T2 > T3 > T4 > T1 > T0. T2 had the highest fecundity rate at 151.7% (*p* < 0.05). 

#### 3.3.3. Effect of Nutritional Flushing on Lambing Traits 

Dietary supplementation increased LR, LS, and LBW, as shown in Table 7. The LR for treatments T2 and T3 were >80 and significantly higher (*p* < 0.05) than other treatments. The lambing rate with no supplementation was 60%. T1 and T2 significantly increased (*p* < 0.05) LS, but T4 had a similar LS to the control. Dietary supplements T1, T3, and T4 tended to increase (*p* < 0.05) litter weight at birth (LBW), but T2 increased LBW significantly (*p* < 0.05). 

## 4. Discussion

Ewes in this study had live weights between 27.10 kg and 28.14 kg. The pasture provided DM of 1.10–1.46 kg/day, which covered the ewes’ DM requirements up to late gestation. However, the pasture provided a CP content of 9.52%, which was insufficient for breeding, mid-gestation, and lambing. It was below CP requirements of 16.1, 13.1, and 14.8, respectively. The pasture could only provide adequate energy for breeding for small ewes weighing up to 30 kg. The energy provided by pasture was insufficient for all ewes > 30 kg in mid-gestation and at lambing, providing 6.9–9.2 MJ/day, which is below the requirement of 11.92–16.32 MJ/day required for mid-gestation and lambing [21]. Under these conditions, flushing the flock with a protein and/or energy-based supplement can improve reproductive performance, as evidenced by numerous studies [22,23,24]. Flushing with protein proves beneficial for flocks on a low-protein diet, such as those grazing on a low-protein pasture. The nutrient composition of the pasture in this study is typical of low-quality pastures with CP < 15%, NDF > 55%, and ADF > 35% [21]. Enset leaf was included in this current study as a supplementary protein source. The findings in this study indicate that the CP content of the enset leaf was 13.21%, which aligns with the results reported by ref. [10] for a mixture of enset leaf lamina and leaf midrib, where it was 130 g/kg DM, and ref. [9], where it was 209 g/kg DM. However, the results did not agree with ref. [25] who reported CP, NDF, ADF, and ash content of 59, 73, 14.5, 20, and 8.5 g/kg DM, respectively. Inconsistent results could be due to varietal differences and differences in the proportion of lamina and midrib at leaf maturity. This implies the importance of selecting enset varieties whose whole leaves contain a CP content of over 7%. As there are several varieties, there may be some that are even more favorable for feeding ruminants, e.g., with a higher CP and lower fiber content. The enset plant has great diversity in southern Ethiopia, and varietal selection for high CP is recommended. 

In terms of BW, the results indicated that flushing may have transformed nutrients into body reserves, resulting in an augmented BW of ewes, consistent with reports from Pakistan [26] that recorded an increase in BW in ewes fed supplemental concentrate during the breeding season. Similar results were reported by refs. [27,28] in Corriedale ewes supplemented with concentrates during breeding. However, the results of ref. [29] on Awassi sheep supplemented with barley contradicted the current findings. The inconsistency of the current results could be linked to differences in the breed of ewes, variations in diet and feeding practices, and the environmental conditions of each experiment. 

The effect of BW and the condition of ewes during mating on production efficiency, primarily through their influence on puberty, is highly significant [30]. According to the results of the present study, flushing during early gestation resulted in an increased trend in the BCS of the ewes, and this effect was sustained through lambing. This may be attributed to the partitioning of nutrients in the feed supplements for weight gain and higher BCS, which later translated into better reproductive performance. Flushed ewes from the T2 and T3 groups attained an optimum BCS of over 3 (on a scale of 1 to 5), resulting in economic benefits due to higher lambing rates (LR) and litter. This is consistent with the findings of ref. [31], who found that Dohne Merino ewes supplemented at mating with 0.45 kg corn grain/head/day had higher BCS (3.2 and 2.9). This was consistent with ref. [32], who reported that conception and lambing rates increased significantly with improving BCS in the Cheviot sheep breed but decreased significantly when BCS was below 2.5. 

The present study found that the onset of estrous with supplemental treatments is consistent with previous research [33], which found that estrous onset in Malpura was 36.6 ± 3.4 h later after sponge removal than in ewes flushed with concentrate supplementation. However, this result was lower than results reported for Farahani ewes (29 h) after synchronization with flurogestone acetate (FGA) sponges when the ewes received 300 g concentrate/day/ewe [30]. Such differences could be attributed to species differences and the amount of supplementation. These findings were almost akin to those of ref. [34], where estrous Yankasa ewes exhibited estrous 33.48 h after synchronization with PGF2α while being fed cottonseed cake supplements. However, the results were not consistent with ref. [35] who reported an onset of estrous in Baladi ewes 18.3 days earlier when fed a concentrate-based diet supplemented with 0.3 mg selenium per kg of diet. This difference could be attributed to breed differences and the timing of flushing. 

In the present study, the duration of estrous observed in Doyogena ewes ranged from 24.66 to 35.46 h. This is similar to previous reports on Kheri ewes, where the duration of estrus was between 28.5 to 30.6 h [36]. In addition, Merino ewes flushed with 600 g alkali-ionophore treated maize/ewe/day [37] and 31.27 Abou-Delik ewes flushed with 300 g barley grain/ewe/day [37] had estrus durations of 33.9 and 31.27 h, respectively, which were comparable to the current study. However, the duration of estrus in the Farahani and Yankasa breeds was shorter (20 and 25.3 h, respectively) when the ewes were flushed with a concentrate mix and cottonseed cake, respectively [30,34]. Moreover, in Malpura ewes fed a concentrate mix, a shorter estrus duration of 22.7 h was reported by ref. [38]. These differences may be due to variations in the type of nutritional flushing employed.

The response rate to estrus in T1 and T2 groups was 100%, which is in line with the results of ref. [39], who found a 100% response rate in Abou-Delik ewes on a flushing diet of 300 g barley grain/head/day) and a 98.33% response rate in Akkaraman ewes flushed with 700 g/ewe/day barley for 35 days. In T3, the response rate was 85.7%, consistent with the findings of ref. [40], where an estrous response rate of 85% was reported in ewes flushed with a balanced mix of 58% ground yellow corn plus 41% ground soybean. However, lower OR rates of 80% were observed in ewes fed 3 kg Egyptian clover [40] and 80% in ewes flushed under traditional management [41]. The observed differences could be attributed to variations in species differences, supplementation levels, and management. Nutritional flushing can be an effective strategy to improve the response rates of estrus and subsequent conception rates, thus enhancing the efficiency of sheep production.

The conception rate of the current study is similar to the findings of a study by ref. [42], which reported a conception rate of 72.7% in Marwari ewes flushed with a 300 g concentrate supplement daily for 21 days. However, this result is lower than that reported by ref. [43]. Breed type and the method and duration of flushing could explain these differences. The treatment groups (T2, T3, T4) showed a 100% conception rate, which is higher than the 66.6% conception rate reported in Awassi ewes fed 500 g concentrate/head/day for three weeks prior to mating [44], and the 52–68% conception rate for Barbarine ewes supplemented with a diet of 0.5 kg of hay and 0.5 kg of concentrate per ewe per day [45]. The amount of supplemented feed may explain the differences. The conception rates of the ewes in the treatment groups (T2 and T3) are consistent with the findings of ref. [46], who reported an 83.3% conception rate for Nari Suwarna ewes fed ragi straw supplemented with concentrate and ref. [47], who reported an 85% conception rate in Barki ewes fed concentrate and bean straw.

The LR observed in the present study was comparable to previous findings by refs. [48,49], where supplementation of ewes led to improved LRs. The LR of the T2 and T3 groups was consistent with the results reported in refs. [37,40], where the LR in ewes was 80% after being flushed with 0.5 kg sesban forage per day and supplemented with 600 g alkali-ionophore treated maize/ewe/day. Additionally, the LR of the T2 and T3 groups was also consistent with ref. [50], who reported an LR of 78% in Ossimi ewes after flushing with concentrates. This could be due to similarities in both protein and energy content which are essential for enhancing reproductive performance. 

The fecundity rate observed in the current study was consistent with results reported by ref. [51], where Barki ewes fed a pelleted concentrate mix and Egyptian clover hay exhibited a fecundity rate of 100%. However, it was lower than the fecundity rate of 200% reported by ref. [52] for Awassi ewes supplemented with 800 g of carrot per ewe per day. The differences in fecundity rate could be attributed to variations in the type and amount of supplemented feed as well as breed differences. The current results contradict the findings of ref. [53], who reported a fecundity rate of 112.5% for Fulbe ewes supplemented with 200 g cottonseed meal/ewe/day. On the other hand, it was higher than the fecundity rate of 75% reported by ref. [54] for ewes flushed under traditional management. Furthermore, they differ from the results reported by ref. [54] who reported fecundity rates of 100%, 134%, and 94%, which could be attributed to differences in climatic variables related to feed availability and management conditions in the studies.

The gestation period observed in the present study was lower than that reported by ref. [40], who found that the gestation period was 167 ± 2.92 and 168 ± 7.72 days in ewe lambs fed 0.5 kg sesban forage. These discrepancies in the gestation period could be attributed to differences in the type of feed, level of supplementation, and management practices during the experimental periods.

Differences in LS (LS) between breeder cooperatives in the same area have been reported by ref. [54]. Within community-based breeding programs (CBBP) in Ethiopia, ewes in Begedamu, Ancha, and Hawora have recorded LS as high as 1.75, 1.70, and 1.61, respectively, while women-only CBBPs have recorded LS as low as 1.29. The high LS of 1.75 is consistent with the results of T2 in the current study, which involved supplementing natural pasture grazing with 250 g EL and 500 g CC, suggesting that farmers within breeder cooperatives are supplementing their ewes to enhance reproduction. The current study found that LS was even higher (*p* < 0.05) at 1.83 when the supplement consisted of 400 g EL and 500 g CC, suggesting that combining enset with concentrate in a ratio of 4:5 is a promising approach to enhance LS in Doyogena ewes.

## 5. Conclusions

The findings of this study suggest that providing Doyogena ewes with increased levels of protein and energy over an extended period can lead to improvements in body weight (BW), body condition score (BCS), pregnancy, and lambing performances. Flushing ewes with a combination of enset leaf and commercial concentrate at a ratio of 4:5 (T2) or 5:4 (T3) for three weeks before and one week after breeding has the potential to increase the conception rate and lambing rate (LR) by 38–42% and 17–24%, respectively, thus improving the economic viability of sheep production. Future research should focus on shortening the flushing period and identifying enset varieties with higher protein and lower fiber content that are better suited for ruminant feed. Additionally, investigating how leaf maturity affects the nutritional value of whole enset leaves could provide further insights.

## Figures and Tables

**Table 1:** Nutritional composition of the basal diet and supplements fed to ewes.

Nutrients	Natural Pasture	Supplements	
		Enset Leaf	Commercial Concentrate
DM (%)	93.56	92.53	92.99
ASH	10.98	6.34	11.30
CP (% DM)	8.72	13.21	17.55
NDF (% DM)	65.85	61.56	33.93
ADF (% DM)	40.23	38.14	19.38
ADL (% DM)	5.94	3.34	6.20
ME (MJ/kg)	6.34	7.74	7.95
IVOMD	45.67	56.31	55.21

DM, dry matter; CP, crude protein; NDF, neutral detergent fiber; ADF, acid detergent fiber; ADL, acid detergent lignin; CC, commercial concentrate; ME, metabolizable energy; IVOMD, in vitro organic matter digestibility; NP, natural pasture.

**Table 2:** Experimental treatments and their nutritional supply to ewes per unit metabolic body weight.

Treatment (T)	Supplemental Diet	Total (% DM)		CP (% DM)		ME (MJ/kg)	
		BW1	BW2	BW1	BW2	BW1	BW2
T0	NP (control)	1.10	1.46	9.52	12.7	6.9	9.2
T1	NP + (250 g EL + 500 g CC/day)	1.78	2.15	15.04	13.4	12.4	14.7
T2	NP + (400 g EL + 500 g CC/day)	1.93	2.29	14.89	14.6	13.5	15.8
T3	NP + (500 g EL + 400 g CC/day)	1.93	2.29	14.68	14.5	13.5	15.8
T4	NP + (500 g EL + 250 g CC/day)	1.78	2.15	14.45	13.4	12.4	14.7

EL, enset leaf; g, gram; NP, natural pasture; CP, crude protein; ME, metabolizable energy; NDF, neutral detergent fiber; BW1, bodyweight of 30 kg; BW2, bodyweight at 40 kg.

**Table 3:** Body weights of Doyogena ewes at the start of the experiment, during breeding, mid-gestation, and lambing.

	Treatments (kg, Means)					
Parameters	T0	T1	T2	T3	T4	*p*-Value
BW-I	27.14	27.70	28.29	28.00	28.14	0.9106
BW-AI	29.14 ^b^	32.86 ^a^	34.14 ^a^	33.07 ^a^	31.86 ^a^	0.0054
BW-P	34.93 ^b^	37.14 ^a^	39.07 ^a^	38.93 ^a^	37.64 ^a^	0.0036
BW-L	31.14 ^c^	34.43 ^ab^	36.14 ^a^	35.57 ^a^	32.79 ^c^	0.0011

BW: Body weight; BWB: Initial body weight; BWAI: Body weight during AI; BWP: Body weight at mid-gestation; BWL: Body weight at lambing; Different superscripts within a row differ at *p* < 0.05.

**Table 4:** Effect of nutritional flushing on body condition score of Doyogena ewes.

	Treatments						
Parameters	T0	T1	T2	T3	T4	SEM	*p*-Value
BCS-I	2.53	2.51	2.56	2.54	2.53	0.17	0.9996
BCS-B	2.57 ^b^	2.90 ^a^	3.07 ^a^	3.00 ^a^	2.79 ^ab^	0.18	0.0100
BCS-G	2.71 ^c^	2.97 ^abc^	3.16 ^a^	3.03 ^ab^	2.86 ^bc^	0.16	0.0232
BCS-L	2.79 ^b^	3.00 ^ab^	3.17 ^a^	3.04 ^ab^	2.83 ^b^	0.13	0.0227

BCS-I: Initial body condition score at the beginning of the study; BCS-B: Body condition score during breeding; BCS-G: Body condition score at mid-gestation; BCS-L: Body condition score at lambing. Different superscripts within a row differ at *p* < 0.05.

**Table 5:** Effects of nutritional flushing on estrous response of Doyogena ewes synchronized with PGF2ἀ.

	Treatments (Means ± SEM)						
Parameters	T0	T1	T2	T3	T4	SEM	*p*-Value
Estrous response rate (%) Onset of estrous (hours)	71.4 ^c^ 46.65 ± 3.77 ^a^	100 ^a^ 35.39 ± 3.93 ^bc^	100 ^a^ 32.43 ± 5.77 ^c^	85.7 ^b^ 34.14 ± 6.08 ^c^	71.4 ^c^ 39.35 ± 4.54 ^b^	0.82542	0.001 0.001
Estrous duration (hours)	24.66 ± 2.53 ^c^	30.33 ± 2.92 ^b^	35.46 ± 3.47 ^a^	34.63 ± 3.26 ^a^	29.04 ± 2.70 ^b^		0.001
N	7	7	7	7	7		

N: Number of ewes in the treatment group; means with different superscripts within a row differ at *p* < 0.05.

**Table 6:** Effects of long-term supplementation on conception, lambing, and fecundity rates in Doyogena ewes.

	Treatments					
Parameters	T0	T1	T2	T3	T4	*p*-Value
N	7	7	7	7	7	
Conception rate (%) Gestation (days)	66.67 ^c^ 151.43 ^a^	80 ^b^ 150.29 ^a^	100 ^a^ 150.00 ^a^	100 ^a^ 149.07 ^a^	100 ^a^ 150.57 ^a^	0.0001 0.9061
Fecundity rate (%)	60 ^e^	100 ^d^	157.1 ^a^	133.3 ^b^	120 ^c^	0.0001

N, number of ewes in the treatment group. Different superscripts within a row differ at *p* < 0.05.

**Table 7:** Effects of nutritional flushing on lambing traits in Doyogena ewes.

	Treatments					
Parameters	T0	T1	T2	T3	T4	*p*-Value
Lambing rate (%) Litter size (mean ± SEM)	60.0 ^d^ 1.50 ± 0.20 ^c^	71.4 ^b^ 1.75 ± 0.11 ^ab^	85.7 ^a^ 1.83 ± 0.06 ^a^	83.3 ^a^ 1.60 ± 0.22 ^bc^	66.67 ^c^ 1.50 ± 0.24 ^c^	0.0001 0.0052
Litter weight (kg, mean ± SEM)	2.73 ± 0.088 ^c^	3.04 ± 0.117 ^bc^	3.39 ± 0.097 ^a^	3.19 ± 0.106 ^ab^	2.93 ± 0.091 ^bc^	0.0021

N: Number of ewes in the treatment group; different superscripts within a row represent statistical significance among treatment groups; NS: not significant; *: significant at (*p* < 0.05) and with the same letter (a, b, or c) are not significantly different.

## Data Availability

Not applicable.
